# Editorial: Biology and pathology of tumor viruses in animals, volume II

**DOI:** 10.3389/fvets.2023.1286103

**Published:** 2023-09-14

**Authors:** Rui M. Gil da Costa, Ana Lúcia Abreu-Silva

**Affiliations:** ^1^Postgraduate Programme in Health Sciences (PPGCS), Federal University of Maranhão (UFMA), São Luís, Brazil; ^2^Molecular Oncology and Viral Pathology Group, Research Center of IPO Porto (CI-IPOP), Health Research Network (RISE)@CI-IPOP, Portuguese Oncology Institute of Porto (IPO Porto), Porto Comprehensive Cancer Center (Porto.CCC), Porto, Portugal; ^3^Laboratory for Process Engineering, Environment, Biotechnology and Energy (LEPABE), Faculty of Engineering, University of Porto, Porto, Portugal; ^4^Associate Laboratory in Chemical Engineering (ALiCE), Faculty of Engineering, University of Porto, Porto, Portugal; ^5^Department of Pathology, State University of Maranhão, São Luís, Brazil

**Keywords:** tumor virus, avian leukemia virus, papillomavirus, pathology, cancer

This second volume of the *Biology and pathology of tumor viruses in animals* Research Topic was built upon the success of the first volume and aimed to proceed the discussion of viral agents involved in tumorigenesis. As with the first volume, researchers from different countries contributed original research that add to the current scientific knowledge in this field, as well as a research article that provides a systematic approach to specific oncoviruses. In this volume, four articles were dedicated to papillomaviruses and one to the bovine leukemia virus.

 Peruchi Fernandes et al. contributed an interesting case report describing and unusual mixed infection of bovine papillomaviruses types 2 and 4 (BPV2 and BPV4) in upper alimentary papillomas in a cow. The authors call the reader's attention to the involvement of those viral types in digestive papillomatosis, even in the absence of *Pteridium* spp. and its carcinogens, which are known to contribute for this kind of lesions (1).

Daraban Bocaneti et al.(a) studied bovine cutaneous fibropapillomas associated with BPV2 and described the expression patterns of metalloproteinases (MMPs) 2, 7, 4 and 14 and their tissue inhibitors (TIMPs) 1 and 2. The authors reported that BPV2-positive fibropapillomas show up-regulation of MMP2, MMP7 and MMP9 and down-regulation of MMP14, TIMP1 and TIMP2. The active forms of MMP2 and MMP9 were detected, and the authors suggest that an imbalance between these MMPs and their TIMPs is implicated in the pathogenesis of bovine cutaneous fibropapillomas associated with BPV2.

The same team contributed a related study soon after. In that study, Daraban Bocaneti et al.(b) described the expression patterns of MMP1, MMP8, MMP13, and TIMP3 in bovine fibropapillomas associated with BPV2. The authors observed an up-regulation of MMP8 and MMP13 and down-regulation of MMP1 and TIMP3 in tumor tissues compared with normal skin, further implicating an imbalance between MMPs and their TIMPs in the pathogenesis of bovine fibropapillomas.

Still regarding papillomaviruses, Medeiros-Fonseca et al. contributed a mini review that provides an update on the current knowledge about canine and feline papillomaviruses. The authors described lesions associated with the 24 known canine papillomavirus (CPV) and six feline papillomavirus (FcaPV) genotypes.

Gomes Pereira et al. reported the prevalence of bovine leukemia virus (BLV) infection in cattle from the Brazilian state of Maranhão. The authors found that approximately 9% of tested animals were positive for BLV and were infected with BLV genotype 6.

Overall, the works included in the second volume of this Research Topic contribute to deepen our knowledge of tumor viruses in animals, including their distribution and mechanisms of pathogenesis.

## Author contributions

RG: Writing—original draft, Writing—review and editing. AA-S: Writing—original draft, Writing—review and editing.
